# Numerical solution of diffusive HBV model in a fractional medium

**DOI:** 10.1186/s40064-016-3295-x

**Published:** 2016-09-22

**Authors:** Kolade M. Owolabi

**Affiliations:** 1 Department of Mathematical Sciences, Federal University of Technology, PMB 704, Akure, Ondo State Nigeria; 2 Institute for Groundwater Studies, Faculty of Natural and Agricultural Sciences, University of the Free State, Bloemfontein, 9300 South Africa

**Keywords:** Disease free equilibrium, Fourier spectral method, Exponential integrator, Fractional reaction–diffusion, Nonlinear PDEs, Numerical simulations, Reproduction number, 34A34, 35A05, 35K57, 65L05, 65M06, 93C10

## Abstract

Evolution systems containing fractional derivatives can result to suitable mathematical models for describing better and important physical phenomena. In this paper, we consider a multi-components nonlinear fractional-in-space reaction–diffusion equations consisting of an improved deterministic model which describe the spread of hepatitis B virus disease in areas of high endemic communities. The model is analyzed. We give some useful biological results to show that the disease-free equilibrium is both locally and globally asymptotically stable when the basic reproduction number is less than unity. Our findings of this paper strongly recommend a combination of effective treatment and vaccination as a good control measure, is important to record the success of HBV disease control through a careful choice of parameters. Some simulation results are presented to support the analytical findings.

## Background

In this paper, we consider in one dimensional space the multicomponent fractional reaction–diffusion system. The fractional derivative equation is obtained by replacing the second-order derivatives in classical *n*-variable reaction–diffusion systems, with order *α* in the interval 0 < *α* < 2. A coupled system of *n* (for *n* ≥ 1, *n* integer) species (population densities or concentrations of chemicals) which interact in a nonlinear fashion and diffuse may be modelled by the *n* system of equations

1 $$\frac{{\partial u_{i} }}{\partial t} = D\frac{{\partial^{\alpha } u}}{{\partial x^{\alpha } }} + F_{i} (u),\quad i = 1,2, \ldots ,n,\,\,t \in [0,T),\,\,T > 0$$

with the initial condition

2 $$u(x,0) = u_{0} (x),\quad i = 1,2, \ldots ,n,$$

Subject to any of the boundary conditions:

- In the case of an infinite system, *x* ∈ (−*∞,∞*), here **R** is a subset of (−*∞,∞*).
- $x \in [0,L],\frac{{\partial u_{i} }}{\partial x}(0,t) = \frac{{\partial u_{i} }}{\partial x}(L,t) = 0,i = 1,2, \ldots ,n,$ no-flux or Neumann boundary condition for a finite system, and
- $x \in [0,L],u(0,t) = u(L,t) = u_{\alpha } ,i = 1,2, \ldots ,n,$ called the Dirichlet or fixed concentration boundary condition, also for a fixed system where $u(x,t) \in R^{n} ,F_{i} :R^{n} \to R$.

The fractional derivative operator

$$\frac{{\partial^{\alpha } u}}{{\partial x^{\alpha } }} = \frac{1}{{\Gamma (1 - \alpha )}}\frac{\partial }{\partial x}\int_{0}^{x} {\frac{u(s)}{(x - s)}ds}$$

(with similar expressions for *u*_*i*_, *i* = 1, 2, … , *n*) is the Riemann–Liouville fractional gradient of order *α*. The diffusion matrix is defined as a diagonal matrix *D* = diag(*D*_1_, *D*_2_, … , *D*_*n*_), and the diffusion coefficients *D*_*i*_ which do not depend on *u* must be positive. *u*(*x*,*t*) is a community of species density. Function *F* accounts for the local kinetics of the system.

For the purpose of simplicity, we may write,

3 $$F_{i} (u) = c_{ii} (u)u_{i} + \sum\limits_{m \ne i} {\gamma_{im} u_{m} } ,$$

where $\gamma_{im} > 0$ for $i \ne m$. The reaction kinetics in (1) is expected to satisfy the following conditions:

1. In $R_{ + }^{n}$ = {(*u*_1_*, u*_2_, … , *u*_*n*_)*|u*_*i*_ > 0|}, the vector field (*F*_1_(**u**), *F*_2_(**u**), … , *F*_*n*_(**u**)) has unstable equilibrium state at **0** = (0, 0, … , 0) and asymptotically stable at **A** = (*A*_1_, *A*_2_, … , *A*_*n*_) with *A*_*i*_ > 0 for *i* = 1, 2, … , *n*.
2. The coefficients
  $$r_{i} = c_{ii} (0) = \mathop {\sup }\limits_{{0 \le u_{i} \le A_{i} ;\forall i = 1,2, \ldots ,n}} [c - ii(u)]$$
  must be finite.

Fractional differential equations known as the differentiation and integration of noninteger order of the form (1) are becoming increasingly used as a modelling tool for diffusive processes associated with sub-diffusion, diffusion and super-diffusion scenarios, and have been applied to an increasing number of situations in biochemistry (Yuste et al. 2004), control (Wang and Zhou 2011; Wang et al. 2012), medicine (Erturk et al. 2011; Hall and Barrick 2008; Otte et al. 2016) and mathematical biology or physics (Atangana 2015; Barkari et al. 2000; Tomovski et al. 2012; Wang and Du 2013). In literature, many definitions of fractional derivatives have been given among which are the definitions by Caputo, Grnwald–Letnikov, Hadamard, Marchaud and Riemann–Liouville are regarded as the most useful definitions (Haghighi et al. 2014; Podlubny et al. 2009; Polyanin and Zaitsev 2004; Yang et al. 2010; Zeng et al. 2014, 2015; Zheng et al. 2015; Zhou 2014). Quite Unfortunate that, similar usages of different definitions give rise to different results (Podlubny 1999).

Over the years, several numerical and analytical methods of solution have been adopted to solve both linear and nonlinear equations. Among which are the homotopy analysis method (Alomari et al. 2009), homotopy perturbation method (He 2005; Yildirim and Sezer 2010), Adomian decomposition method (Hassan 2008; Ray 2009), multistep differential transform method (Li Ding and Lin-Jiang 2013; Erturk et al. 2011; Jiang et al. 2012), Kansa method (Chen et al. 2010), finite difference and tau methods (Celik and Duman 2012; Pang and Sun 2012; Saadatmandi and Dehghan 2011; Sausa 2009; Su et al. 2010) to mention a few. In the context of this paper, the spatial complexity of the domain imposes geometric constraints on the transport processes on all length scales, which can be measured as temporal correlations on all the time scales. Hence, we propose the study of fractional hepatitis B Virus (HBV) reaction–diffusion system in sub-diffusive (0 < *α* < 1), diffusive (*α* = 1) and super-diffusive (1 < *α* < 2) scenarios, using the Fourier spectral method in space to remove the stiffness issue associated with the spatial fractional derivative in a finite but large domain size *L*. For the temporal discretization, we employ higher-order exponential time differencing scheme.

In “Basic definitions and numerical techniques for fractional diffusion problems” section, we provide some required basic information and definitions of fractional calculus, we also formulate Fourier spectral method for multicomponents fractional-in-space reaction-diffusion system. We present fractional HBV model, and examine the main system for both local and global stability in “Main model” section. Numerical experiment of the full nontrivial result is provided in “Numerical simulations” section. The final section concludes the paper.

## Basic definitions and numerical techniques for fractional diffusion problems

Two important tasks are done in this section. The first one is to present some of the basic required information and definitions that guide the solution of fractional diffusion equations. The second task is centered on the introduction of Fourier spectral methods as an efficient and easy-to-implement for the integration of fractional-in-space reaction–diffusion systems.

### Basics definitions of fractional calculus

#### **Definition 1**

If *f*(*x*) ∈ [*a*, *b*] and *a* < *x* < *b*, then the Riemann–Liouville fractional integral is given as:

$$I_{\alpha + }^{\alpha } f(x) = \frac{1}{{\Gamma (\alpha )}}\int_{a}^{x} {\frac{f(t)}{{(x - t)^{1 - \alpha } }}} dt.$$

#### **Definition 2**

For *α* ∈ (0, 1),

$$D_{\alpha + }^{\alpha } f(x) = \frac{1}{{\Gamma (1 - \alpha )}}\frac{d}{dx}\int_{a}^{x} {\frac{f(t)}{{(x - t)^{\alpha } }}} dt$$

is known as the Riemann–Liouville fractional integral of order *α* (Pindza and Owolabi 2016; Saadatmandi and Dehghan 2011; Zeng et al. 2014; Zheng et al. 2015) where

$$\Gamma (\alpha ) = \int_{0}^{\infty } {s^{\alpha - 1} e^{ - s} } ds,\quad \alpha > 0.$$

#### **Definition 3**

For −1 < *α* < *n*, *n* ∈ *N*, *x* > 0, and $f \in C_{ - 1}^{n}$, is the Caputo fractional derivative of order *α* is defined as:

$$D_{x}^{\alpha } f(x) = \frac{1}{{\Gamma (n - \alpha )}}\int_{0}^{x} {(x - t)^{n - \alpha - 1} f^{(n)} (t)} dt.$$

#### **Definition 4**

For *n* − 1 < *α* < *n* (Haghighi et al. 2014; Ortigueira 2006; Samko et al. 1993)

$$\frac{{\partial^{\alpha } }}{{\partial |x|^{\alpha } }}u(x,t) = - C_{\alpha } (_{0} D_{x}^{\alpha } +_{0} D_{L}^{\alpha } )u(x,t)$$

Is the Riesz fractional derivative, where $\alpha \ne 1,C_{\alpha } = \frac{1}{{2\cos \left( {\frac{\pi \alpha }{2}} \right)}}$ and

$$\begin{aligned}_{0} D_{x}^{\alpha } u(x,t) & = \frac{1}{{\Gamma (n - x)}}\frac{{\partial^{n} }}{{\partial x^{n} }}\int_{0}^{x} {\frac{u(\xi ,t)}{{(x - \xi )^{\alpha + 1 - n} }}} d\xi , \\_{0} D_{L}^{\alpha } u(x,t) & = \frac{{( - 1)^{n} }}{{\Gamma (n - x)}}\frac{{\partial^{n} }}{{\partial x^{n} }}\int_{L}^{x} {\frac{u(\xi ,t)}{{(x - \xi )^{\alpha + 1 - n} }}} d\xi . \\ \end{aligned}$$

Primarily, the Caputo fractional differential equation computes an ordinary differential equation (ODE), followed by a fractional integral to obtain the desired order of fractional derivative, but in the reverse order we compute the Riemann–Liouville fractional differential system. The Caputo fractional differential equation permits the inclusion of traditional initial and boundary conditions in the problem formulation. These two operators coincide in the case of homogeneous initial condition. For details geometric and physical interpretation for fractional calculus for both the Riemann–Liouville and Caputo types, readers are referred to the classical books (Kilbas et al. 2005; Podlubny 1999).

#### **Theorem 1**

*Consider a one*-*component fractional reaction*–*diffusion model*

4 $$D_{t}^{\eta ,v} u(x,t) = \mu {}_{x}D_{\vartheta }^{\alpha } u(x,t) - \rho u(x,t) + \sigma (x,t)$$

*where μ, t* > 0*, x* ∈ **R***, α, η,*$\sigma$, *ν are real and positive parameters subject to the constraints* 0 < *α* ≤ 2*, |ϑ|* < min(*α,* 2 − *α*)*, and*$D_{t}^{\eta ,v}$*is the generalized Riemann*–*Liouville fractional derivative operator given by*

5 $$D_{a + }^{\eta ,v} u(x,t) = I_{a + }^{v(n - \eta )} \frac{{\partial^{n} }}{{\partial t^{n} }}\left( {I_{0 + }^{(1 - v)(n - \eta )} u(x,t)} \right) = I_{a + }^{{v(n - \eta )(D_{0 + }^{\eta + vn - \eta v} u(x,t))}}$$

*with the conditions*

$$I_{0 + }^{(1 - v)(2 - \eta )} u(x,0_{ + } ) = f(x),\frac{d}{dt}\left( {I_{0 + }^{(1 - v)(2 - \eta )} u(x,0_{ + } )} \right),\mathop {\lim }\limits_{|x| \to \infty } u(x,t) = 0,$$

*where* 1 < *η* ≤ 2, 0 ≤ *ν* ≤ 1*, ρ* > 0 *is a constant with the kinetic (or reaction) term.*${}_{x}D_{\vartheta }^{\alpha }$*is called the Riesz*–*Feller space fractional derivative of order α, skewness ϑ is symmetry given in terms of the Fourier transform*

6 $$F\{ {}_{x}D_{\vartheta }^{\alpha } fx;k\} = - \varpi_{\vartheta }^{\alpha } (k)f^{ * } (k),$$

*where*$\varpi_{\vartheta }^{\alpha } (k) = \left| k \right|^{\alpha } e^{i(signk)} \frac{\vartheta \pi }{2}$, 0 < *α* ≤ 2*, |ϑ|,* min(*α,* 2 − *α*)*, μ is the diffusion coefficient and*$\sigma$(*x, t*) *is a term that belongs to the area of reaction*–*diffusion function. The solution of* (4) *govern by the above conditions is*

7 $$\begin{aligned} u(x,t) & = \frac{{t^{\eta + \mu (2 - \eta ) - 2} }}{2\pi }\int_{ - \infty }^{\infty } {e^{ - ikx} f^{*} (k)G_{\eta ,\eta + \mu (2 - \eta ) - 1} \left[ { - t^{n} (\rho + \eta_{\alpha }^{\vartheta } (k))} \right]} dk \\ & \quad + \frac{{t^{\eta - \mu (\eta - 2) - 1} }}{2\pi }\int_{ - \infty }^{\infty } {e^{ - ikx} g^{*} (k)G_{\eta ,\eta - \mu (\eta - 2)} } \left[ { - t^{\eta } (\rho + \eta_{\alpha }^{\vartheta } (k))} \right]dk \\ & \quad + \frac{1}{2\pi }\int_{ - \infty }^{\infty } {} \int_{0}^{t} {\xi^{\eta - 1} } \sigma^{*} (k,t - \xi )e^{ - ikx} \left[ {G_{\eta ,\eta } \left( { - \xi^{\eta } [\rho + \eta \varpi_{\alpha }^{\vartheta } (k)]} \right)dkd\xi } \right]. \\ \end{aligned}$$

#### *Proof*

For the time variable, we apply Laplace transform w.r.t. time *t*, also for the space variable, we find the Fourier transform w.r.t. *x* using Laplace parameter *s* and Fourier parameter *k*, subject to the initial conditions and Eq. (6) with the Laplace transform of (5) given as

$$L[D_{a + }^{\eta ,v} u(x,t);s] = s^{\eta } \bar{u}(x,s) - \sum\limits_{k = 0}^{n - 1} {s^{s - k - v(n - \eta )} } \frac{{\partial^{k} }}{{\partial t^{k} }}\left( {I^{(1 - v)(n - \eta )\,} u(x,0^{ + } )} \right),$$

for *n* − 1 < *η* ≤ *n, n ∈ u,* 0 ≤ *ν* ≤ 1, we then transform the given equation into the form

$$s^{\eta } \bar{u}^{*} (k,s) - s^{1 - v(2 - \eta )} f^{*} (k) - s^{v(\eta - 2)} g^{*} (k) = - \mu \varpi_{\alpha }^{\vartheta } (k)\bar{u}^{*} (k,s) - \rho \bar{u}^{*} (k,s) + \bar{\sigma }^{*} (k,s),$$

where * is the Fourier transform w.r.t. *x* with Laplace and Fourier parameters *s* and *k*. By convention we use (.) to denote the Laplace transform of (***·***). So, solving for $\bar{u}^{*} (k,s)$ we obtain

8 $$\bar{u}^{*} (k,s) = \frac{{s^{1 - v(2 - \eta )} f^{*} (k) + s^{v(\eta - 2)} g^{*} (k) + \bar{\sigma }^{*} (k,s)}}{{s^{\eta } + \mu \varpi_{\alpha }^{\vartheta } (k) + \rho }}.$$

We take the inverse Laplace transform of Eq. (8) and following the result of Saxena et al. (2006), we have

$$L^{ - 1} \left[ {\frac{{s^{\psi - 1} }}{{a + s^{\alpha } }};t} \right] = t^{\alpha - \psi } G_{\alpha ,\alpha - \psi + 1} ( - at^{\alpha } ),$$

where the real part of the parameters are positive, bear in mind that

9 $$\begin{aligned} \bar{u}^{*} (k,t) & = f^{*} (k)t^{\eta + v(2 - \eta ) - 2} G_{\eta ,\eta + \mu (2 - \eta ) - 1} \left[ { - t^{n} \left( {\rho + \mu \varpi_{\alpha }^{\vartheta } (k)} \right)} \right] \\ & \quad + g^{*} (k)t^{\eta + v(2 - \eta ) - 1} G_{\eta ,\eta + \mu (2 - \eta )} \left[ { - t^{\eta } (\rho + \mu \varpi_{\alpha }^{\vartheta } (k))} \right] \\ & \quad + \int_{0}^{t} {\xi^{\eta - 1} } \sigma^{*} (k,t - \xi )\,\left[ {G_{\eta ,\eta } \left( { - \xi^{\eta } [\rho + \mu \varpi_{\alpha }^{\vartheta } (k)]} \right)} \right]\xi , \\ \end{aligned}$$

which by inverse Fourier transform, Eq. (9) becomes

10 $$\begin{aligned} u(x,t) & = \frac{{t^{\eta + \mu (2 - \eta ) - 2} }}{2\pi }\int_{ - \infty }^{\infty } {e^{ - ikx} f^{*} (k)G_{\eta ,\eta + \mu (2 - \eta ) - 1} \left[ { - t^{n} (\rho + \eta_{\alpha }^{\vartheta } (k))} \right]} dk \\ & \quad + \frac{{t^{\eta - \mu (\eta - 2) - 1} }}{2\pi }\int_{ - \infty }^{\infty } {e^{ - ikx} g^{*} (k)G_{\eta ,\eta - \mu (\eta - 2)} } \left[ { - t^{\eta } (\rho + \eta_{\alpha }^{\vartheta } (k))} \right]dk \\ & \quad + \frac{1}{2\pi }\int_{ - \infty }^{\infty } {} \int_{0}^{t} {\xi^{\eta - 1} } \sigma^{*} (k,t - \xi )e^{ - ikx} \left[ {G_{\eta ,\eta } \left( { - \xi^{\eta } [\rho + \eta \varpi_{\alpha }^{\vartheta } (k)]} \right)dkd\xi } \right], \\ \end{aligned}$$

as the required result. □

### Numerical techniques for fractional reaction–diffusion equations

Spectral algorithms are considered to be extremely valuable for generating numerical schemes in almost every areas of applied mathematics, because it has no excuse to differentiate poorly but possesses the ability to yield spectrally accurate fractional or spatial derivatives. We can always guarantee of getting good and efficient working codes, when spectral methods is coupled with Fast Fourier transforms and implement with high-level language like Matlab. In one component, we use the integrating factor technique to Fourier transform of (1) to get

11 $$U_{t} (X_{x} ) = - (X_{x}^{\alpha } )U(X_{x} ,t) + F[f(u(x,t))],$$

where *U* is the Fourier transform of species *u*(*x*, *t*). This implies that,

$$F[u(x,t)] = U(X_{x} ,t) = \int\limits_{ - \infty }^{\infty } {u(x,t)e^{{ - i(X_{x} x)}} dx} .$$

We let $\varOmega^{\alpha } = (X_{x}^{\alpha } )$ to explicitly remove the issue of stiffness in the fractional derivative term, and set $U = e^{{ - \varOmega^{\alpha } t}} \bar{U}$, so that

12 $$\partial_{t} \bar{U} = e^{{ - \varOmega^{\alpha } t}} F[f(u)]$$

Now, in practice, we discretize spatial domain by considering *N*_*x*_ the equispaced points direction of *x*. We employ the discrete fast Fourier transform (DFFT; Owolabi and Patidar 2015) so that (12) becomes a system of ODEs

13 $$\partial_{t} \bar{U}_{i} = e^{{ - \varOmega_{i}^{\alpha } t}} F[f(u_{i} )],$$

where *u*_*i*_ = *u*(*x*_*i*_) and $\varOmega_{i}^{\alpha } = X_{x}^{\alpha } (i)$. The boundary conditions can be clamped at extremes of the domain. At this point, we have transformed the system to ODEs, more importantly, the spatial derivative and the associated stiffness are gone. We can employ any higher-order explicit solver.

By using the standard notation of order *s* Runge–Kutta (RK) schemes, with time step Δ*t*, we can advance from *t*_*n*_ = *n*Δ*t* to *t*_*n*+1_ = (*n* + 1)Δ*t* for the ODE

$$u_{t} = f(u,t):u_{n + 1} = \sum\limits_{i = 1}^{s} {c_{i} k_{i} }$$

where

$$k_{i} =\Delta tf\left( {t_{n} + a_{i}\Delta t,u_{n} + \sum\limits_{j = 1}^{i - 1} {b_{j} k_{i} } } \right).$$

Here, we apply the general explicit Runge–Kutta scheme to (13) for $\bar{U}_{i}$. For brevity, we denote *μ*_*i*_ as *k′* in $\overline{{U_{i} }}$ and set the replacement variable as

$$\bar{\mu }_{i} = \mu_{i} \exp ( - \varOmega^{\alpha } t_{n} ).$$

Finally, we write the *s*-stage RK scheme as

14 $$U_{n + 1} = \exp ( - \varOmega^{\alpha }\Delta t)\left[ {U_{n} + \sum\limits_{i = 1}^{s} {c_{i} \bar{\mu }_{i} } } \right],$$

with modified term

$$\bar{\mu }_{i} = \exp (\varOmega^{\alpha } a_{i}\Delta t)\Delta tF\left[ {f(F^{ - 1} (U_{{n + \alpha_{i} }} ))} \right]$$

and *U* values at intermediate step

$$U_{{n + \alpha_{i} }} = \exp ( - \varOmega^{\alpha } a_{i}\Delta t)\left[ {U_{n} + \sum\limits_{j = 1}^{i - 1} {b_{ij} \bar{\mu }_{i} } } \right].$$

This implies that we work entirely in the spectral domain and invert a transform to recover *u*. It should be mentioned that once the stiffness is removed, one can rapidly and accurately advance in time with any explicit higher-order time-solvers, see for instance (Kassam and Trefethen 2005; Owolabi and Patidar 2014) for details.

For the second time stepping solver, we formulate and utilize the modified Krogstad (2005) version of the Cox and Matthews scheme (Cox and Matthews 2002), which we denoted here for brevity as ETDRK4.

15 $$\begin{aligned} U_{n + 1} & = U_{n} e^{{{\text{L}}h}} + h[4\varphi_{3} ({\text{L}}h) - 3\varphi_{2} ({\text{L}}h) + \varphi_{1} ({\text{L}}h)]{\text{F}}(u_{n} ,t_{n} ) \\ & \quad + 2h[\varphi_{2} ({\text{L}}h) - 2\varphi_{3} ({\text{L}}h)]{\text{F}}(\mu_{2} ,t_{n} + h/2) \\ & \quad + 2h[\varphi_{2} ({\text{L}}h) - 2\varphi_{3} ({\text{L}}h)]{\text{F}}(\mu_{3} ,t_{n} + h/2) \\ & \quad + h[\varphi_{3} ({\text{L}}h) - 2\varphi_{2} ({\text{L}}h)]{\text{F}}(\mu_{4} ,t_{n} + h). \\ \end{aligned}$$

with the stages *μ*_*i*_ given as

$$\begin{aligned} \mu_{2} & = U_{n} e^{{{\text{L}}h/2}} + ({\text{L}}h/2)\varphi_{1} ({\text{L}}h/2)F(u_{n} ,t_{n} ) \\ \mu_{3} & = U_{n} e^{{{\text{L}}h/2}} + ({\text{L}}h/2)[\varphi_{1} ({\text{L}}h/2) - 2\varphi_{2} ({\text{L}}h/2)]F(u_{n} ,t_{n} ) \\ & \quad + h\varphi_{2} ({\text{L}}h/2)F(\mu_{2} ,t_{n} + h/2), \\ \mu_{4} & = U_{n} e^{{{\text{L}}h}} + h[(\varphi_{1} ({\text{L}}h) - 2\varphi_{2} ({\text{L}}h)]F(u_{n} ,t_{n} ) + 2h\varphi_{2} ({\text{L}}h){\text{L}}(\mu_{3} ,t_{n} + h), \\ \end{aligned}$$

The functions *φ*_*i*_, are given as

$$\phi_{1} (z) = \frac{{e^{z} - 1}}{z},\quad \phi_{2} (z) = \frac{{e^{z} - 1 - z}}{{z^{2} }},\quad \phi_{3} (z) = \frac{{e^{z} - 1 - z - z^{2} /2}}{{z^{3} }}$$

where L = $\frac{{\partial^{\alpha } u}}{{\partial x^{\alpha } }}$ is the fractional derivative operator with order *α*, and **N** is the Fourier transform that accounts for the nonlinear reaction functions. Readers are referred to Cox and Matthews (2002) and Owolabi and Patidar (2016a, b) for details derivation and stability of the family of exponential time differencing schemes.

At this point, we need to check the performance of the fractional Fourier transform technique in conjunction with both the classical fourth-order Runge–Kutta (Owolabi and Patidar 2014) and the fourth-order exponential time-differencing Runge–Kutta schemes. Here we consider (1) in one component and set the reaction term to zero, so that the given multicomponents system reduces to fractional diffusion equation.

**Fig. 1:**
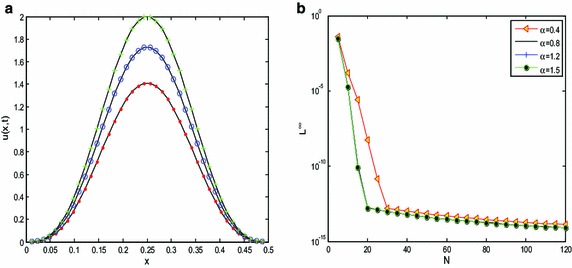
**a** Comparison of the numerical and analytical solutions of the fractional diffusion equation at *α* = 1.5, *D* = 0.5, obtained at instances of *t* = 2 (*dots*), *t* = 3 (*circles*) and *t* = 4 (*crosses*). The *thick lines* represent the analytical solutions. **b** Convergence of the fractional Fourier method at instances of *α* at final time *t* = 1

In Fig. 1, we report the L^*∞*^-norm error defined as

16 $$L^{\infty } (N) = \frac{{\left\| {u - \overline{u} } \right\|_{\infty } }}{{\left\| u \right\|_{\infty } }} = \mathop {\hbox{max} }\limits_{1 \le j \le N} \frac{{\left| {u_{j} - \overline{{u_{j} }} } \right|}}{{u_{j} }},$$

where $\bar{u}$ and *u* are the approximate and exact solutions, and *N* represents the computational grids number, on *x* ∈ [0, 0.5] at *t* = 1 and initial condition *u*_0_(*x*) = exp[− 10*x*^2^*/*(1 − *x*^2^)]. The reference solution is taken by evaluating the fractional diffusion equation using 212 Fourier modes. It is proper to say that regardless of fractional power *α*, our approach (Fourier transform) is able to attain a spectral convergence up to machine precision.

Timing results (independent of fractional power *α*) are given in Table 1 for comparison between both schemes. Indication here is that, the ETDRK4 displays a better performance when used in conjunction with the Fourier spectral method, especially when *N* is large. In terms of efficiency and accuracy, Fourier methods when compared to low-order methods have proven to be advantageous relative to memory requirement, computationally efficient and fast in execution times. The result obtained in Table 1 justifies the reason for abandoning the RK4 scheme in the main computations.

**Table 1:** The relative error for fractional diffusion equation for various values of discretization, at *D* = 0.5 and *t* = 1

N	*N* = 64	*N* = 128	*N* = 256	*N* = 512
RK4 ETDRK4	1.960e−06 1.6391e−08	4.9539e−06 2.0899e−08	7.5637e−06 2.9017e−08	1.5528e−05 4.7984e−08
Ratio	72.9669	237.0400	260.6644	323.6079

## Main model

Over the years, mathematical modelling has become an important tool in the application areas medical and life sciences to address some of the health challenging problems that are not approachable experimentally. Hepatitis B is commonly referred to as a life threatening infectious disease caused by the hepatitis B virus (HBV) which leads to inflammation or causing serious damage to the liver. According to World Health Organization’s (WHO) 2002 data report, over 2000 million people have been effected, more than 350 million individuals remain chronically infected and carriers of the virus. An estimated population of 4 million people are considered as acute clinical cases of the virus.

Research have shown that children and adolescents are most vulnerable to the disease than adults due to exposure which may show some clinical symptom and have a higher percentage chance of being acutely infected. Based on report, about a quarter of chronic infected individuals die of liver cancer annually. As a result, hepatitis B is known to be one of the most common viral source of cancer in the world nowadays. Hence, HBV infection is a disease of global health and its prevalence varies from one region to the other.

A lot of researchers have worked on HBV in the past, among which are the notable papers of Medley et al. (2001) where compartmentalized model was used to describe the spread of the disease. Almost a decade later, Zou et al. (2010) worked on the modified version of the model (Medley et al. 2001). They develop a model to explore the impact of vaccination and other controlling measures of HBV infection. Their model has simple dynamical behavior which has a globally asymptotically stable disease-free equilibrium when the basic reproduction number *R*_0_ < 1, and a globally asymptotically stable endemic equilibrium when *R*_0_ > 1. In the year 2014, Kimbir et al. (2014) give an extension to the earlier report in Zou et al. (2010) by including the treatment of chronically infected HBV carriers, it was also suggested in their report that the acute infected individuals are not subjected to antiviral treatment due to natural recovery. Wiah et al. (2015) employed a nonlinear extended deterministic model to address the impact of immigration on the population spread of HBV infection with acute and chronic infected carriers.

In this work, we present a deterministic model consisting of coupled nonlinear fractional partial differential equations of order *α*. This new model provides an extension of the models discussed earlier by Zou et al. (2010, 2015). The model population consists of five local kinetics broken into the susceptible individuals (*U*_1_), exposed class (*U*_2_) which are infected but yet to be infectious, acute infection individuals (*U*_3_), chronic HBV class (*U*_4_) and temporary protective immunity referred to as the recovered individuals (*U*_5_). The fractional reaction–diffusion system is given as

17 $$\frac{{\partial U_{i} }}{\partial t} - D_{i} \frac{{\partial^{\alpha } U_{i} }}{{\partial x^{\alpha } }} = F_{i} (U_{i} ),\quad i = 1,2, \ldots ,5,\,\,t > 0$$

where *u* = (*u*_1_, *u*_2_, … , *u*_*n*_) is a vector of concentration or density for interacting species at position *x* and time *t*, and *F*_*i*_, *i* = 1, … , 5, are the local reaction terms. The terms *D*_*i*_ > 0*, i* = 1, … , 5 are the diffusion coefficients. The initial densities are expected to be non-negative and the problem (17) is confined by imposing the appropriate choice of boundary conditions. The boundary conditions are taken as zero flux

18 $$\left. {\frac{{\partial U_{1} }}{\partial x}} \right|_{x = 0} = \left. {\frac{{\partial U_{1} }}{\partial x}} \right|_{x = L} = 0$$

with similar relations for the *U*_*i*_, *i* = 2, … , 5. The initial data are taken as in Eq. (2) in the form of some small perturbations from the uniform solution *:

19 $$U_{1} (x,0) = U_{i}^{*} + U_{i0} (x),\quad \left| {U_{i0} (x)} \right| < < \left| {U_{i}^{*} } \right|$$

with analogous expressions for the remaining species. Subject to the following coupled reaction kinetics

20 $$\begin{aligned} F_{1} (U_{1} ,U_{2} , \ldots ,U_{5} ) & = \gamma - \omega (U_{3} + \psi U_{4} )U_{1} - (\tau + \phi )U_{1} , \\ F_{2} (U_{1} ,U_{2} , \ldots ,U_{5} ) & = \omega (U_{3} + \psi U_{4} )U_{1} - (\delta + \varepsilon + \tau )U_{2} , \\ F_{3} (U_{1} ,U_{2} , \ldots ,U_{5} ) & = \delta U_{2} - [(1 - \varphi )\sigma - \tau ]U_{3} , \\ F_{4} (U_{1} ,U_{2} , \ldots ,U_{5} ) & = (1 - \varphi )\sigma U_{3} - (\tau - \beta )U_{4} , \\ F_{5} (U_{1} ,U_{2} , \ldots ,U_{5} ) & = \phi U_{1} + \varepsilon U_{2} - \tau U_{5} \\ \end{aligned}$$

where *γ* represents the rate of recruitment into a susceptible individuals, *ω* stands for the transmission rate of disease from one infection class to another. The HBV induced rate is given by *β*, while *τ* is the natural death rate. Parameters *δ*, *σ* are the progressive rate from the exposed (*U*_2_) to acute (*U*_3_) infection individuals and acute to chronic infection class (*U*_4_) respectively. The natural recovery rate for the exposed individuals for the latent HBV is denoted by *ϵ*, while *ψ* ≫ 1 is the transmission rate multiplier. Finally, *ϕ* and *φ* are the respective vaccination success rate for the class *U*_1_ and treatment success rate for (*U*_3_) infected class.

### Stability analysis of the disease free equilibrium (DFE) point

In this section, we analyze the local stability of the disease-free equilibrium (DFE). It is the stability of at DFE that can guarantee a biologically meaningful results. Here we assume that the disease variables *U*_2_ = *U*_3_ = *U*_4_ = 0. If otherwise, the disease will persist and put the whole population of susceptible individuals into serious danger.

The basic reproduction number, commonly denoted as *R*_0_, gives the total number of secondary infections that an average infectious class will induce given that the rest of the population is susceptible. By using the notation in Pang et al. (2010), we denote the emergence of new infection by *F*, and the transfer of individual from one class to another by *V*. The endemic equilibrium dynamics in the region $(U_{1} > 0,U_{2} > 0,U_{3} > 0,U_{4} > 0,U_{5} > 0) = (\hat{U}_{1} ,\hat{U}_{2} ,\hat{U}_{3} ,\hat{U}_{4} ,\hat{U}_{5} )$, do not correspond to biologically meaningful results since it encourages the spread of HBV disease. Hence, we do not capture the endemic equilibrium results in the analysis. For all possible parameter values, the spatially homogeneous stationary solution of model (17) with kinetics (20) has a disease free equilibrium point $\hat{E} = (\gamma /(\tau + \phi ),0,0,0,\tau \phi /(\tau + \phi )\tau )$, we define the reproduction number as *R*_0_ = (*FV*^−1^), where

$$F = \left( {\begin{array}{*{20}c} 0 & {\frac{\omega \tau }{\tau + \varphi }} & {\frac{\psi \omega \tau }{\tau + \varphi }} \\ 0 & 0 & 0 \\ 0 & 0 & 0 \\ \end{array} } \right),\quad V = \left( {\begin{array}{*{20}c} {\delta + \varepsilon + \tau } & 0 & 0 \\ { - \delta } & {\tau + (1 - \phi )\sigma } & 0 \\ 0 & {(\phi - 1)\sigma } & {\tau + \beta } \\ \end{array} } \right).$$

After some algebraic manipulations, we obtain the average value of the expected number of secondary cases produced by a single infected individuals

21 $$R_{0} = \frac{{\left[ {\tau + \beta + (1 - \phi )\psi \sigma } \right]\omega \tau \delta }}{(\tau + \beta )(\delta + \varepsilon + \tau )(\tau + \varphi )((1 - \phi )\sigma + \tau )}$$

#### **Theorem 2**

*The disease free equilibrium point*$\hat{E} = (\gamma /(\tau + \phi ),0,0,0,\tau \phi /(\tau + \phi )\tau )$*is locally asymptotically stable for the spatially homogeneous stationary solution of model* (17) *with kinetics* (20) *if R*_0_ < 1*, and unstable if otherwise.*

#### *Proof*

The Jacobian matrix of the spatially homogeneous stationary solution of model (17) with kinetics (20) at point $\hat{E}$ is given by

22 $$J_{{\hat{E}}} = \left( {\begin{array}{c@{\quad}c@{\quad}c@{\quad}c@{\quad}c} { - (\varphi + \tau )} & 0 & { - \frac{\omega \tau }{\varphi + \tau }} & { - \frac{\psi \omega \tau }{\varphi + \tau }} & 0 \\ 0 & { - (\delta + \varepsilon + \tau )} & {\frac{\omega \tau }{\varphi + \tau }} & {\frac{\psi \omega \tau }{\varphi + \tau }} & 0 \\ 0 & \delta & { - (\tau + (1 - \phi )\sigma )} & 0 & 0 \\ 0 & 0 & {\tau + (1 - \phi )\sigma } & { - (\beta + \tau )} & 0 \\ \varphi & \varepsilon & 0 & 0 & { - \tau } \\ \end{array} } \right)$$

From the characteristics equation of (22), we have two negative eigen values $\lambda_{1} = - \tau ,\lambda_{2} = - (\tau + \phi )$. After substitution, we have the rest of the characteristic polynomial given as

23 $$[{\mathbf{A}} - \lambda {\mathbf{I}}] = (a_{11} - \lambda )[(a_{22} - \lambda )(a_{33} - \lambda )] - a_{12} [a_{21} (a_{33} - \lambda )] \, + a_{13} (a_{21} a_{32} ) = 0$$

where **A**_*ij*_, *i*, *j* = 1, 2, 3 is a 3 × 3 matrix, and **I** identity matrix. From (23), we obtain the characteristic equation $X(\lambda ) = \lambda_{3} - (a_{11} a_{22} a_{33} )\lambda_{2} + (a_{11} a_{22} + a_{11} a_{33} + a_{22} a_{33} + a_{12} a_{21} )\lambda - a_{12} a_{21} a_{33} - a_{12} a_{22} a_{33} - a_{13} a_{21} a_{32} = 0$.By adopting the Routh–Hurwitz stability conditions of the linear differential equations, system (17) with kinetics (20) is stable for the disease free equilibrium point $\hat{E}$ if: (1) the roots *r*_0_, *r*_1_, *r*_2_, *r*_3_ are positive with negative real parts, where *r*_3_ = 1, *r*_2_ = *a*_11_*a*_22_ + *a*_11_*a*_33_, *r*_1_ = (*a*_11_*a*_22_ + *a*_11_*a*_33_ + *a*_22_*a*_33_ + *a*_12_*a*_21_), *r*_0_ = *a*_12_*a*_21_*a*_33_ − *a*_12_*a*_22_*a*_33_ − *a*_13_*a*_21_*a*_32_ (2) *r*_1_*r*_2_ − *r*_0_*a*_3_ > 0. We verified from the coefficients that *r*_0_ > 0, *r*_1_ > 0, *r*_2_ > 0, *r*_3_ > 0 and *r*_1_*r*_2_ − *r*_0_*a*_3_ = − *R*_0_ > 1 which implies that *R*_0_ < 1. Hence we complete the proof since *R*_0_ < 1 which shows that the disease free equilibrium point is locally asymptotically stable. □

### Global stability of the diseases free equilibrium point

Here, we are concerned with the global stability of DFE point. We adopt a similar technique to the proof of Theorem 2.

#### **Theorem 3**

*The disease free equilibrium point*$\hat{E} = (\gamma /(\tau + \phi ),0,0,0,\tau \phi /(\tau + \phi )\tau )$*is globally asymptotically stable for the spatially homogeneous stationary solution of model* (17) *with kinetics* (20)*, if R*_0_ < 1.

#### *Proof*

Let *G* be a Lyapunov function given in the form

24 $$G(Ui, \, t) = PU2 + U3 + QU4,\quad i = 1(1)5.$$

On differentiating (24) and substitute the spatially homogeneous version of model (17) with kinetics (20) into it at $\hat{E}$, we have

25 $$G^{{\prime }} \le (\delta - P(\psi + \delta + \tau ))U_{2} + \left( {P\frac{\omega \tau }{\varphi + \tau } + Q(1 - \phi )\sigma } \right)U_{3} + \left( {P\frac{\omega \psi \tau }{\varphi + \tau } - Q(\tau + \beta )} \right)U_{4} ,$$

on substituting for $P = \frac{\delta }{\tau \varepsilon \delta }$ and $Q = \frac{\psi \omega \tau \delta }{(\tau \varepsilon \delta )(\tau + \beta )(\varphi + \tau )}$ in (15) we get

$$\begin{aligned} G^{{\prime }} & \le (\sigma (1 - \phi ) + \tau )\left( {\underbrace {{\frac{[\tau + \beta + (1 - \phi )\psi \sigma ]\omega \tau \delta }{(\tau + \beta )(\delta + \varepsilon + \tau )(\tau + \varphi )((1 - \phi )\sigma + \tau )} - 1}}_{{R_{0} }}} \right), \\ & \le (\sigma (1 - \phi ) + \tau )(R_{0} - 1) \le 0. \\ \end{aligned}$$

It follows from the point $\hat{E} = (\gamma /(\tau + \phi ),0,0,0,\tau \phi /(\tau + \phi )\tau )$ that *G* = 0 since the derivatives of *Ui* = 0, *i* = 2, 3, 4. This implies that as *U*_*i*_ → 0, *i* = 2, 3, 4, also *U*_1_ → *γ*/(*τ* + *ϕ*) and *U*_5_ → *τϕ*/(*τ* + *ϕ*)*τ* as *t* → *∞*. So the DFE point is globally asymptotically stable if the inequality is satisfies the condition *R*_0_ < 1, but unstable if *R*_0_ > 1. The proof is completed. □

## Numerical simulations

In this section, we start to simulate numerically the solution of the spatially homogeneous system (17) with kinetics (20) using the numerical techniques formulated in “Basic definitions and numerical techniques for fractional diffusion problems” section above to substantiate our analytical findings.

### Non-diffusive example

We consider the set of parameters

26 $$\phi = 2.5,\gamma = 2.0,\omega = 0.5,\psi = 2.5,\delta = 0.5,\varepsilon = 1.5,\varphi = 0.75,\beta = 2.5$$

with reasonable initial populations (millions)

27 $$U1(0) = 95.5,U2(0) = 2.0,U3(0) = 3.0,U4(0) = 1.5,U5(0) = 1.0.$$

For the set of ecological parameters, we realized that the conditions given in Theorems 2 and 3 for non-diffusive system are satisfied for the disease free-equilibrium state.

**Fig. 2:**
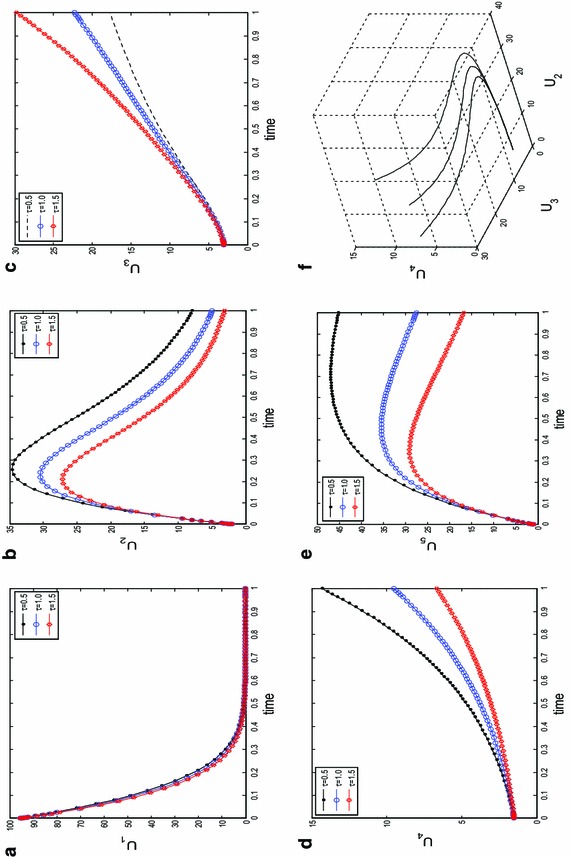
Time series results for the spatially homogeneous version of system (17) with kinetics (20), obtained at different instants of *τ* and final time *t* = 1. Other parameters are fixed in (26)

**Fig. 3:**
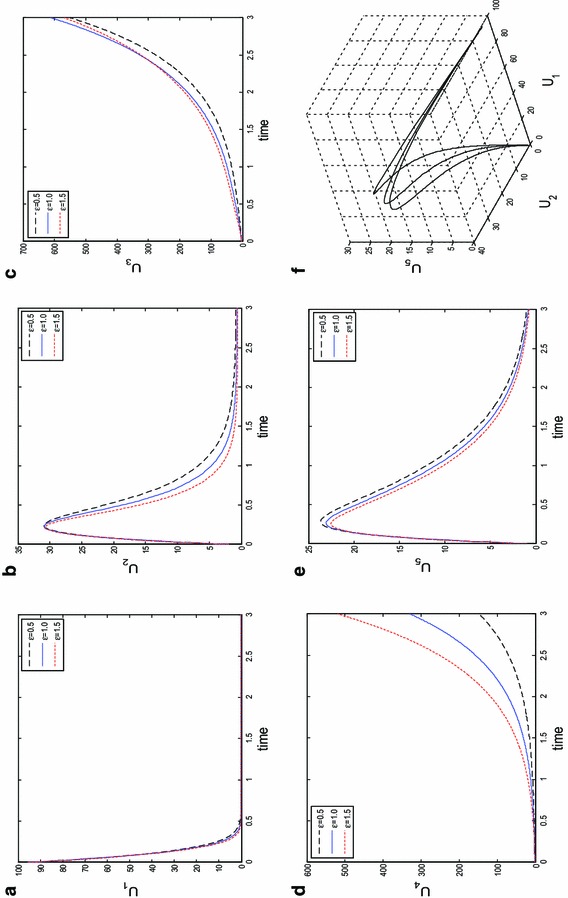
Time series results for the spatially homogeneous version of system (17) with kinetics (20), obtained at different instants of *ϵ* and final time *t* = 3. Other parameters are fixed in (26)

In Fig. 2, we simulate the non-diffusive system numerically at different instances of *tau* to study the behaviour of the species with time (year) *t* = 1. It was observed only the class of individuals *U*_1_, *U*_2_, *U*_5_ could actually be free of HBV as time is progressed. Clearly, those in the classes *U*_3_ and *U*_4_ which corresponds to the number of acute and chronic individuals will continue to live and spread the virus as years roll-by until the entire population is endermic. Similarly in Fig. 3, we fixed all parameters and initial conditions as in (26) and (27), and simulate with different instances of *ϵ*. It is obvious, despite the necessary measures such as treatments and lots of sensitization programs put in place for those in the acute and chronic class to get recovered with increasing time *t* = 3, the number of casualties is increasing. This is evident on the axis containing the population profiles of *U*_3_ and *U*_4_. Species attractor at *t* = 3 and instances of *ϵ* is given in panel (f).

**Fig. 4:**
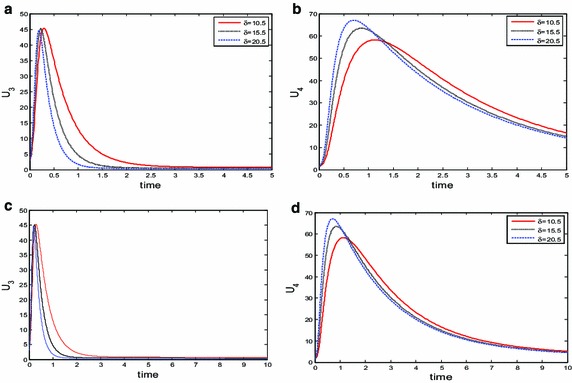
Behaviour of the acute and chronic individuals to treatment at instances *δ* and time *t*. Parameters are: *τ* = 0.5, *β* = 0.05 at *t* = 5 and *t* = 10 for species *U*_3_ and *U*_4_. Both species respond to treatment at the control parameters level with respect to time. Other parameters are fixed in (26)

Since it is not possible to completely wipe out both *U*_3_ and *U*_4_ classes in model (or in the population). Then, the question of ‘what can be done?’ sets in. In the context of this paper, we came out with the opinion of varying some of the parameters, the correct choice of parameters that will put control to the spread of HBV is attained by reducing the values of *τ* from 1.5 to 0.5 and that of *β* from 2.5 → 0.05 to enable the class of individuals *U*_4_ respond to treatment over time. To checkmate the spread of HBV for the group *U*_3_, we need to increase parameter value *δ* from 1.5 to 10.5 and above. These assertion is evident in Fig. 4.

### Fractional reaction–diffusion example

Here, we simulate the whole system in the presence of diffusion with fractional power index *α*. We now illustrate through numerical experiments with Neumann boundary conditions, a multiple coexistence steady states in the fractional-in-space reaction–diffusion model (17) with kinetics (20). The boundary conditions are clamped the the extreme points of spatial domain of size [0, *L*], *L* = 20. We compute the initial conditions as:

$$\begin{aligned} {\text{U}}_{1} &= 95.5*{\text{ones}}({\text{N}},1);{\text{U}}_{2} = 2*{\text{ones}}({\text{N}},1);{\text{U}}_{3} = 3*{\text{ones}}({\text{N}},1); \hfill \\ {\text{U}}_{4} &= 1.5*{\text{ones}}({\text{N}},1);{\text{U}}_{5} = 1*{\text{ones}}({\text{N}},1); \hfill \\ \end{aligned}$$

where *N* is the number of discretization.

**Fig. 5:**
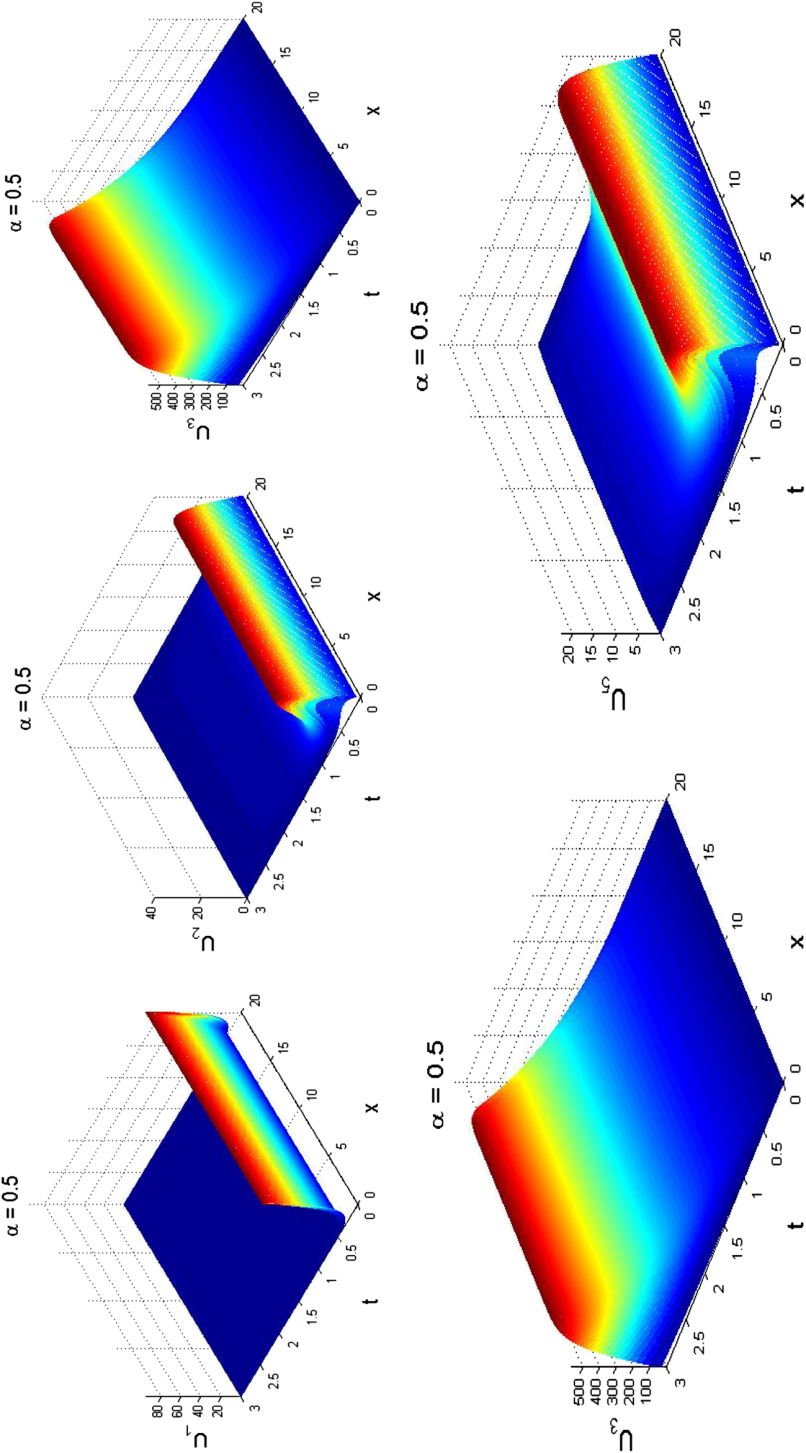
Surface plots for fractional reaction–diffusion system (17) with kinetics (20) at sub-diffusive scenario when *α* = 0.5 and *t* = 3. The dynamics obtained are similar to those presented in Figs. 2 and 3. Parameters are given in (26)

We fixed parameters as:

28 $$\begin{aligned} &\{ \phi = 2.5,\tau = 1.5,\gamma = 2.0,\omega = 0.5,\psi = 2.5,\delta = 1.5,\varepsilon = 0.5, \hfill \\ &\quad \varphi = 0.75,\beta = 2.5,D1 = 0.2,D2 = 0.5,D3 = 0.25,D4 = 0.1,D5 = 0.5\} \hfill \\ \end{aligned}$$

to obtain the surface plots displayed in Fig. 5. Obviously, the behaviour of the species correspond to those in Figs. 2 and 3, though the amplitudes differ.

**Fig. 6:**
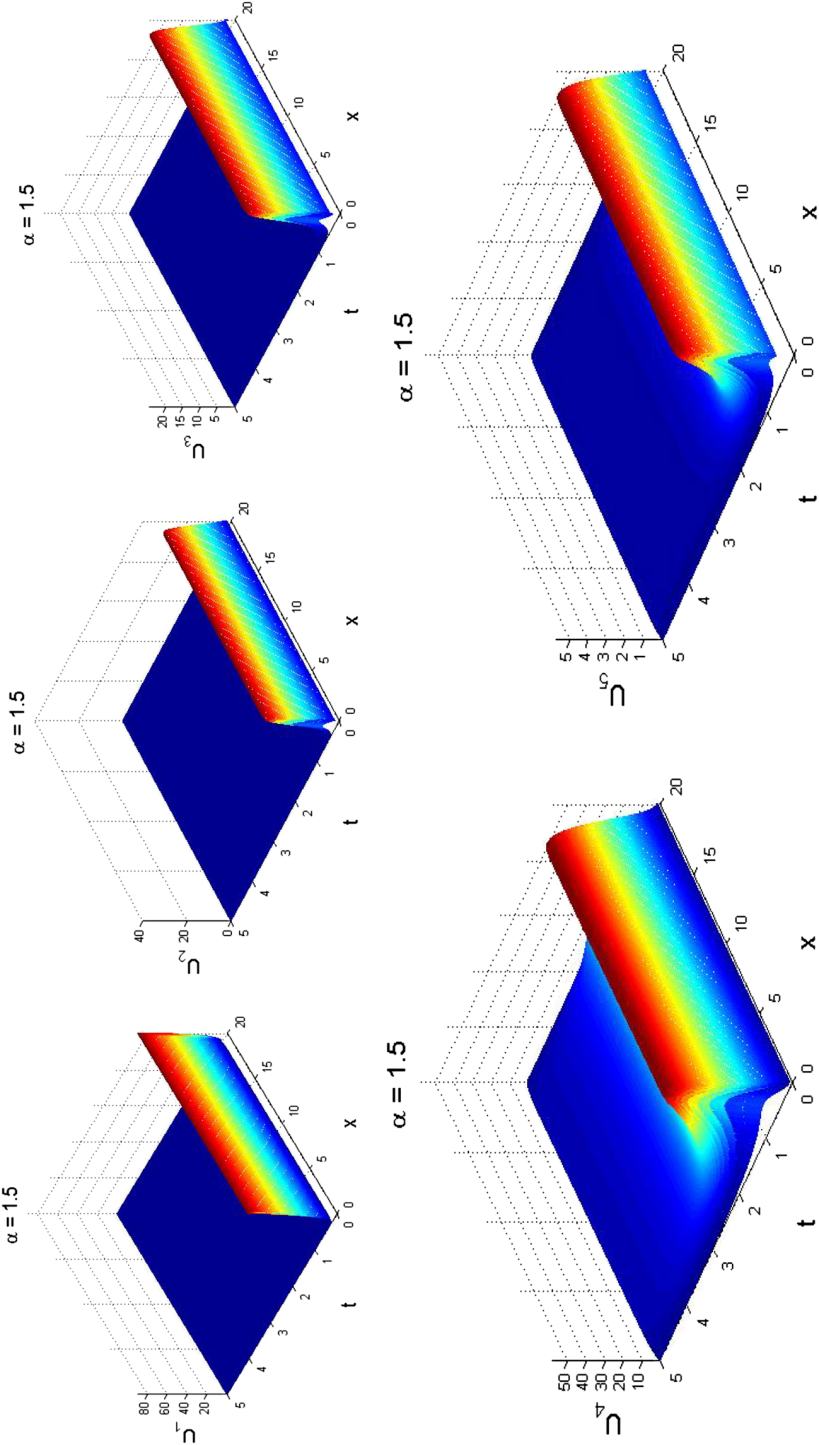
Surface plots showing super-diffusive results for fractional reaction–diffusion system (17) with kinetics (20) obtained at fractional derivative index *α* = 1.5 and *t* = 5 to mimic results obtained in Fig. 4

We repeat the parameter values used in Fig. 4 to obtain the surface plots displayed in Fig. 6 for the super-diffusive case, when *α* = 1.5. Clearly, all the species are disease free when necessary vaccinations and treatments (drugs) are administered over time. The risen amplitudes indicating high cases of HBV drop-down or tend to zero to show that the spread of HBV has significantly reduced, as shown in Fig. 6. By the computer simulation of the fractional reaction–diffusion system, we have given evidence that hepatitis B virus model and control can be studied in fractional scenarios. Our findings in this paper strongly recommend a combination of effective treatment and vaccination as a good control measure is important to record the success of HBV disease control as done via parameters *τ*, *β* and *δ*.

## Conclusion

In this paper, a mathematical model for investigating the hepatitis B virus disease in fractional medium is derived. The model disease free equilibrium state is analyzed. We established via theorems that the model disease-free equilibrium is both locally and globally asymptotically stable, if the basic reproduction number is less than unity. Our aim is to examine the behaviour of diffusive fractional reaction–diffusion model in sub-diffusive and super-diffusive scenarios, derive efficient and reliable numerical techniques. By the computer experiment of the fractional reaction–diffusion system we have given enough evidence that numerical solution in the diffusive (fractional) scenario, at 0 < *α* < 2 is practicably the same as in the case of non-diffusive case when applied to model Hepatitis B virus system. Our findings in this work strongly recommend a combination of effective treatment and vaccination as a good control measure is important to record the success of HBV disease control. It should be noted that the methodology presented in this paper can be applied to model other physical phenomena in higher dimensions.
